# London Dispersion in Confined Chiral Ion-Pair Asymmetric Catalysis: From Background Interaction to Design Principle

**DOI:** 10.1021/acs.accounts.6c00299

**Published:** 2026-07-21

**Authors:** Lorenzo Baldinelli, Giovanni Bistoni

**Affiliations:** † 9309 Istituto CNR di Scienze e Tecnologie Chimiche “Giulio Natta” (CNR-SCITEC), Perugia 06123, Italy; ‡ Department of Chemistry, Biology and Biotechnology, University of Perugia, Perugia 06123, Italy

## Abstract

As molecular systems of increasing complexity come to dominate the frontiers of chemistry, biology, and materials science, the temptation grows to bypass mechanistic understanding in favor of correlations (whether empirical or learned) that capture trends without illuminating their origins. Yet, for systems governed by dense networks of noncovalent interactions, correlations without causation offer limited transferability. The deeper challenge lies in forging an understanding that is at once quantitatively rigorous and chemically intuitive: principles rooted in the physics of these interactions, capable of explaining why a system behaves as it does and of guiding its rational redesign.

We believe that extracting such transferable insights from the accurate characterization of noncovalent interactions in complex systems is a central objective of modern computational chemistry. This challenge intensifies as molecular architectures grow in size and flexibility, entering a regime in which the tools of small-molecule quantum chemistry and the sampling demands of biomolecular simulations must converge. Here, we examine an especially challenging case study: harnessing London dispersion in confined asymmetric catalysis.

London dispersion is among the most pervasive yet least exploited forces in asymmetric catalysis. In enzymes, precisely shaped active sites achieve extraordinary selectivity by confining substrates within pockets rich in noncovalent contacts. Confined chiral catalysts emulate this strategy: their enclosed active sites shape the conformational space of catalyst–substrate assemblies multiplying short-range contacts, enhancing a contribution whose role in stereocontrol grows with the degree of confinement.

Recognizing and quantifying this role, however, demands computational strategies that match the complexity of the systems involved: large, flexible supramolecular assemblies in which competing enantiomeric pathways are separated by fractions of a kcal·mol^–1^. This Account shows how such strategies enable quantitative prediction of enantioselectivity and reveal London dispersion as an often overlooked yet engineerable interaction in confined asymmetric catalysis.

Using imidodiphosphorimidate (IDPi) catalysts as the central case studies, we demonstrate that an accurate description of their systems requires treating key intermediates and transition states as conformational ensembles rather than single structures, where the role of the solvent adds a further layer of supramolecular complexity. With ensembles ranked to identify the arrangements that are thermally accessible under reaction conditions, electronic structure analysis enables the disentangling of the noncovalent interactions that discriminate between competing pathways.

Building on the combination of quantitative accuracy and interaction-level insight, we examine recent representative case studies that illustrate how London dispersion plays a crucial role in shaping function in chiral ion-pair (CIP) catalysis. These examples reveal recognition modes reminiscent of enzymatic catalysis—from rigid lock-and-key complementarity, in which the catalyst pocket provides a fixed dispersion landscape that selectively rewards one substrate orientation, to induced-fit scenarios in which the catalytic cavity reorganizes to maximize dispersive contacts with the bound substrate, without however compromising excessively intracatalyst dispersion. We further show how dispersion involving the surrounding environment, including solvent–catalyst–substrate interactions, can introduce additional energetic bias and shift selectivity.

Overall, our work emphasizes that London dispersion can be considered a structurally programmable lever for selectivity, encoded through the rational codesign of catalyst, substrate, and solvent.

## Graphical abstract

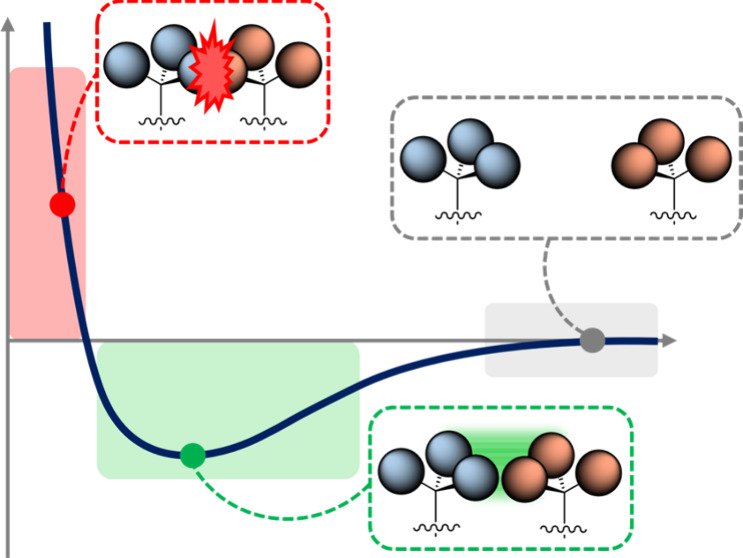

## Key References

-

Yepes, D. ; Neese, F. ; List, B. ; Bistoni, G. Unveiling the Delicate Balance of Steric and Dispersion Interactions in Organocatalysis Using High-Level Computational Methods. J. Am. Chem. Soc. 2020, 142, 3613–3625 31984734 10.1021/jacs.9b13725PMC7307905.^1^ This work shows that quantitative stereochemical predictions in confined asymmetric catalysis require ensemble-based treatment of reaction intermediates and transition states, highlighting the decisive role of London dispersion in controlling selectivity.
-

Baldinelli, L. ; De Angelis, F. ; Bistoni, G. Unraveling Atomic Contributions to the London Dispersion Energy: Insights into Molecular Recognition and Reactivity. J. Chem. Theory Comput. 2024, 20, 1923–1931 38324509 10.1021/acs.jctc.3c00977.^2^ The Atomic Decomposition of London Dispersion (ADLD) was introduced, providing a rigorous framework for atom-resolved identification of dispersion contributions across complex molecular and supramolecular systems.
-

Kayal, R. ; Baldinelli, L. ; Harden, I. ; Neese, F. ; Bistoni, G. Understanding and Quantifying the Impact of Solute-Solvent van Der Waals Interactions on the Selectivity of Asymmetric Catalytic Transformations. Chem. Sci. 2025, 16, 2700–2709 39802687 10.1039/d4sc04329dPMC11718370.^3^ Our computational results show that solute–solvent van der Waals interactions can decisively bias enantioselectivity. In addition, the Molecular Dispersion Potential (MDP) was introduced as a field-based descriptor to visualize and rationalize dispersion effects in real space.
-

Baldinelli, L. ; Lerda, S. ; Kayal, R. ; Neese, F. ; De Angelis, F. ; Bistoni, G. Deciphering Asymmetric Induction in Photoredox Catalysis by Chiral Counteranions. ACS Catal. 2026, 16, 766–774 41503059 10.1021/acscatal.5c07578PMC12772447.^4^ This work demonstrates that London dispersion governs stereocontrol in photoredox catalysis mediated by chiral counteranions.

## 1 Introduction

Achieving high selectivity is essential across pharmaceuticals, fine chemicals, and sustainable synthesis. In modular catalysts, this selectivity can be engineered by modifying well-defined structural elements.^5^ As competing pathways are often separated by free-energy gaps of only fractions of a kcal·mol^–1^, even subtle structural modifications can decisively shift selectivity.^6,7^ One effective way to amplify these small energetic differences is through confinement:^8^ shaping the active site of the catalyst into a restricted space that controls substrate orientation and enhances the influence of noncovalent interactions.

A growing class of confined organocatalysts now merges features of enzymes with small-molecule catalysis.^9,10^ These systems possess enzyme-like structural complexity while maintaining well-defined active sites and engineerable reaction environments. A paradigmatic realization is the structural evolution of chiral phosphoric acid (CPA)-derived catalysts,^11,12^ particularly the development of increasingly confined architectures by List and co-workers. This progression moves from the relatively open triisopropylphenyl-substituted (TRIP) scaffold^13,14^ to the deeply confined imidodiphosphorimidate (IDPi) architecture,^15,16^ whose confined chiral cavity delivers high stereoinduction even for relatively small substrates.^1,8,17,18^ These catalysts have become defining architectures in CIP organocatalysis^19^ and underpin asymmetric counteranion-directed catalysis (ACDC)^20,21^ by enforcing selectivity through confined ion pairing. More recently, the ACDC framework has been extended to photoactivated radical processes in asymmetric counteranion-directed photoredox catalysis (ACPC),^22^ underscoring the versatility of such confined architectures across distinct mechanistic regimes.

From its earliest formulations, it was clear that ion pairing cannot be reduced to a simple Coulombic attraction.^20^ While electrostatics remain the dominant prerequisite for ion-pair formation, an increasing body of mechanistic evidence indicates that selectivity arises from the dense network of multiple noncovalent interactions operating within these complex supramolecular assemblies.^1,23–25^

Understanding and ultimately predicting how this noncovalent network encodes selectivity^26,27^ is beyond the reach of experiment alone. Yet these confined catalytic systems pose a distinctive challenge for theory, sitting at the interface between two traditionally separate computational domains.^10^ Enzymes, being large and flexible, have traditionally been studied using molecular-dynamics-based methods, which efficiently explore configuration space but rely on force fields of limited accuracy. Small-molecule catalysts, by contrast, are routinely treated with high-level *ab initio* and density functional methods capable of resolving sub-kcal·mol^–1^ or even sub-kJ·mol^–1^ electronic energy differences, but these approaches presuppose that the relevant stationary points can be identified within a manageable conformational landscape. Confined organocatalysts demand both exhaustive sampling to capture the conformational degrees of freedom that modulate the interaction network and high-level electronic-structure methods to resolve the subtle energetic balances that govern selectivity (Figure 1).

**Figure 1:**
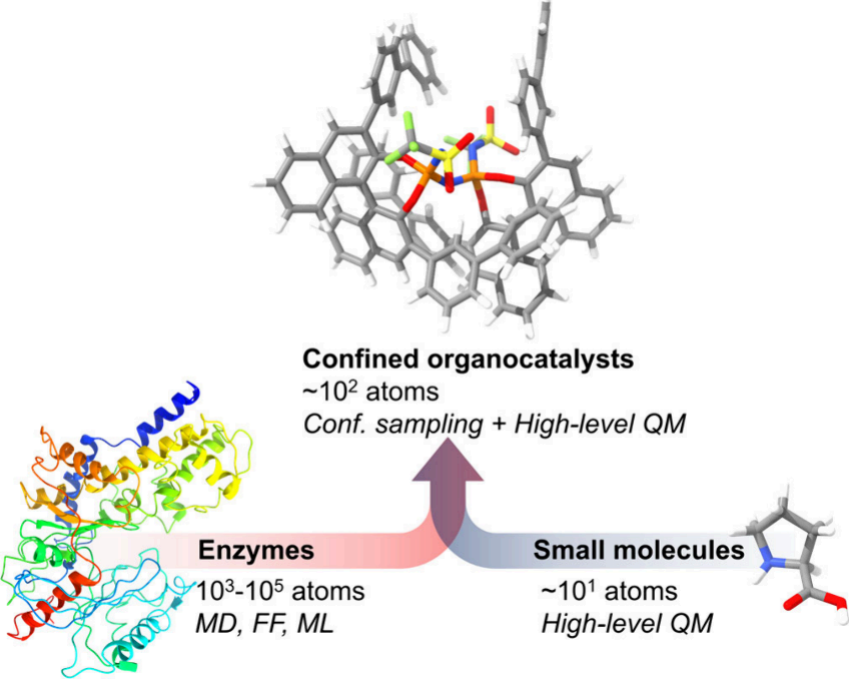
Computational challenges across the catalysis spectrum. Left: Hexokinase, originally used to explain the induced-fit model, exemplifies enzyme catalysis, requiring extensive molecular dynamics (MD) only accessible with force fields (FF) or machine learning (ML) methods. Right: l-Proline represents small-molecule organocatalysis, treated with high-level quantum chemistry (QM) to achieve chemical accuracy. Center: Confined catalysts, such as IDPi, demand both conformational sampling combined with high-level QM methods to resolve selectivity.

Well-tailored protocols integrating these two requirements are therefore indispensable to obtain reliable geometries and energetics.^4,25,28^ Equally important are robust analysis tools that connect structure to function,^2,29^ moving beyond simply reproducing experimental selectivities toward understanding how noncovalent interactions arise from molecular architecture, respond to targeted modifications, and ultimately shape the observed properties. Classical steric and electrostatic descriptors provide an essential starting point in this direction, rationalizing accessibility, ion pairing, and global organization.^30,31^ Yet when competing states are nearly degenerate, these models alone cannot resolve the intricate noncovalent network that ultimately governs selectivity. It is precisely here that the London dispersion assumes a central role. Long regarded as a ubiquitous but secondary contribution, dispersion becomes decisive in confined catalytic assemblies, where its stabilization scales with the number and spatial organization of contacts within the catalytic pocket.^1,25,32–34^ Small structural changes, such as the substitution of a single aryl group or the reorientation of an alkyl fragment, can reshape dispersion patterns and tip the energetic balance between competing pathways. Crucially, this sensitivity renders dispersion *engineerable*: it can be modulated through the same modular design elements that make these catalysts so powerful.^34,35^

In this Account, we trace our efforts to understand and exploit the interplay among confinement, complexity, and London dispersion in confined CIPs. IDPi-based architectures serve as the primary platform.^1,4,25,36^ Case studies involving the binaphthyl-allyl-tetrasulfone (BALT) counteranion (more rigid and lacking a defined recognition pocket) broaden the analysis to other ACDC architectures,^1,2^ and direct comparisons with IDPi highlight how the degree of confinement impacts dispersive contributions to enantiocontrol. Across catalyst, substrate, and solvent, we show how structural modifications reorganize dispersive interaction networks and predictably bias enantioselectivity,^2–4,25^ establishing these three components as a coupled supramolecular system that must be addressed as a whole. Concepts and tools presented here^2,3,37,38^ advance dispersion as a transferable design principle for reactivity and selectivity, whose impact can be amplified by confinement to a decisive and programmable energetic contribution.

## 2 Sampling Crowded Energy Landscapes

The dual computational challenge outlined in the Introduction of combining exhaustive conformational sampling with high-level electronic-structure accuracy becomes immediately concrete when one attempts to predict enantioselectivity in confined CIPs. Selectivity emerges from the balance of noncovalent interactions within the catalytic pocket, but *which* balance is operative depends critically on *which* molecular arrangements are thermally populated under reaction conditions. The organization of the catalyst–substrate assembly—and, in principle, the surrounding solvent—is not static: multiple geometries may be thermally accessible, and defining the chemically relevant conformational ensemble is the indispensable first step.

Many of these degrees of freedom are weakly coupled to the formal reaction coordinate, yet they can significantly shift the relative free energies. This applies to transition states as much as to intermediates, making systematic conformational sampling essential for a reliable selectivity prediction. The importance of this requirement was recognized early by Seguin and Wheeler,^31^ whose analysis of BALT-based CIPs combined MM-level sampling and constrained QM reoptimization. The field has responded with a rapidly growing toolbox. Approaches such as AARON,^39^ ACE,^40^ and Q2MM,^41^ together with platforms including CatVS^42^ and Virtual Chemist,^43^ exemplify a broader attempt to shift toward automated, ensemble-based selectivity prediction. In parallel, semiempirical and machine-learning approaches, including hybrid strategies, aim to extend the practical boundaries of chemical space exploration and catalyst screening.^44–47^

Our own approach, first introduced in the context of IDPi-based ACDC,^1^ placed the entire sampling and refinement procedure within a consistent QM framework (Figure 2a).

**Figure 2:**
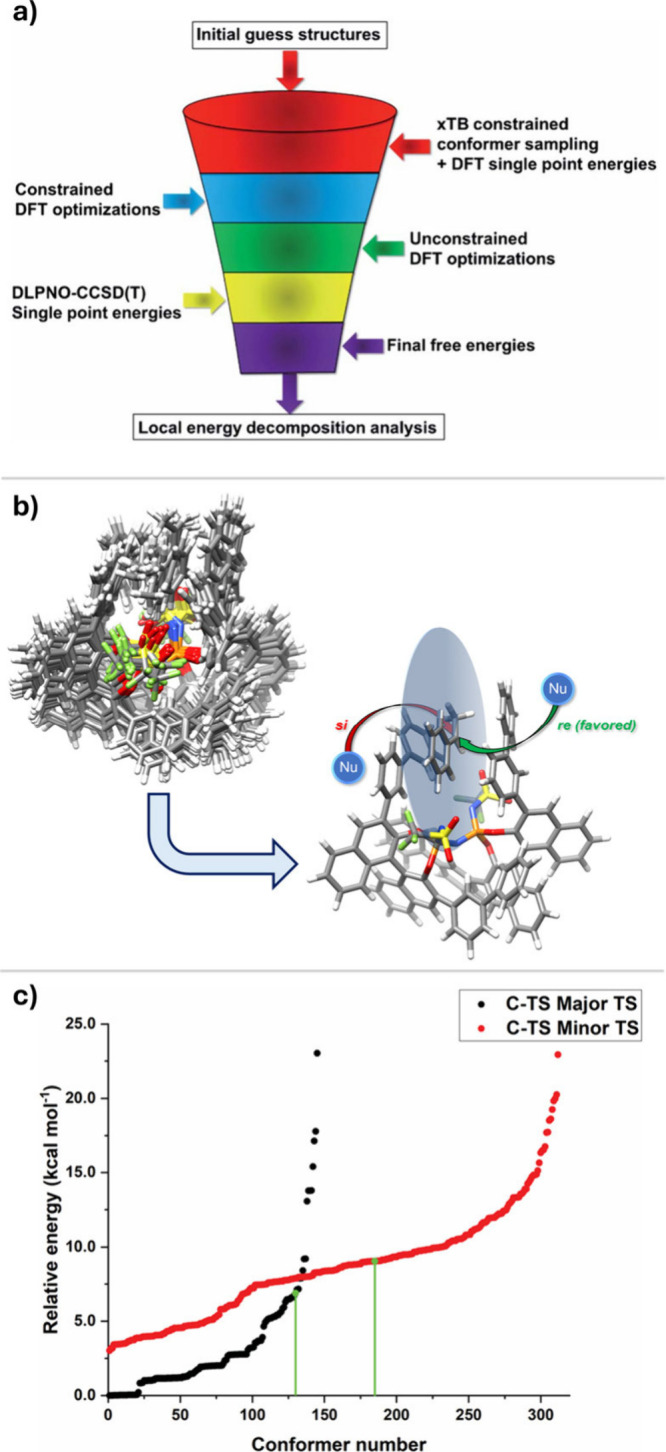
(a) Filtration strategy for identifying thermally accessible intermediates and transition states; applied to both enantiomeric pathways for selectivity calculations. Adapted from ref 25 under CC BY 3.0. (b) Energy refinement of the conformational ensemble identifies the lowest-energy ion-pair arrangement, whose pocket organization controls nucleophile face exposure. Adapted with permission from ref 36. Copyright 2023 The American Association for the Advancement of Science. (c) Relative energies obtained from constrained geometry optimizations for transition states leading to the major (black) and minor (red) enantiomeric products in the intramolecular hydroalkoxylation study in ref 25. Transition-state energies originally discussed in ref 18 are shown in green, illustrating the importance of systematic conformational sampling. Adapted from ref 25 under CC BY 3.0.

Initial exploration is performed using semiempirical tight-binding methods using tools such as CREST^48^ and GOAT^49^ to generate structurally diverse ensembles. The objective at this stage is exhaustive configurational coverage rather than final energy accuracy. Similar conformers can be removed through structural comparison and clustering to define a representative subspace. The selected ensemble is energy-sorted at a density functional theory (DFT) level to identify the low-energy regions of the landscape. In particularly challenging cases, further refinement at higher levels of theory, such as DLPNO–CCSD­(T),^50^ as implemented in ORCA,^51^ can be necessary to secure a robust energetic ordering. This filtration approach provides a reliable structural basis for understanding how geometric organization and noncovalent interactions determine the catalytic outcomes.

A prerequisite for meaningful ensemble generation, however, is that it samples the right species. In the free IDPi anion, intramolecular dispersion tends to favor compact conformations that partially shield the active site.^1^ Upon ion pair formation, accommodation of the cation can drive the catalyst toward geometries that differ substantially from the free-anion minima, opening the pocket to maximize complementarity with the bound substrate. The extent of this reorganization is expected to grow with the flexibility of the catalyst,^25^ suggesting that precise structural information about the isolated anion may become increasingly uninformative as the system grows in complexity. Accordingly, in this class of systems, the most relevant structural insights are obtained from studies that directly target CIPs or transition states. Two case studies illustrate this concept: one at the CIP level and one at the transition state.

What ensemble refinement reveals at the CIP level is illustrated in Figure 2b, for the asymmetric S_N_1 platform we investigated in ref 36. The lowest-energy geometry (Figure 2b) exhibits a distinct pocket organization that governs nucleophile positioning and controls which face of the benzylic cation is sterically accessible, thereby biasing the subsequent S_N_1 attack. This well-defined facial accessibility is not apparent in certain higher energy conformers.This underscores why identifying all thermally accessible structures is critical to defining the stereochemically relevant intermediate.

At the transition-state level, our intramolecular hydroalkoxylation computational study reveals an analogous picture.^25^ Although the forming C–O bonds constrain the core topology of the transition state, the surrounding ion-pair scaffold retains substantial flexibility. Systematic conformational sampling revealed hundreds of transition-state conformers within narrow energy windows, differing primarily in their supramolecular organization. Our protocol identified multiple low-lying structures below those originally proposed in ref 18, as shown in Figure 2c.

These examples converge on a conclusion: high-level theory applied to the wrong geometries converges, with increasing precision, toward the wrong answer. Of course, this does not diminish the central role of high-level methods, but highlights the need to couple them with systematic sampling strategies. Importantly, when multiple conformers lie close in energy, selectivities should be derived from Boltzmann-weighted ensembles. However, in our experience, the effective selectivity is often dominated by the lowest-energy transition-state conformer of each pathway. Within practical computational limits, accurately ranked ensembles therefore represent best practice for reliable mechanistic interpretation. Looking ahead, the frontier lies in generating such ensembles systematically across catalyst, substrate, and solvent libraries—shifting the question from *why* this catalyst gives this ee, to *which* catalyst, for which substrate, and in which medium. Reaching this goal will probably require the cooperation of complementary approaches—semiempirical methods for broad conformational exploration, high-level theory for energetic accuracy, and machine learning for bridging the two across large chemical spaces. Machine learning, in particular, remains largely underexplored in this context: models trained on interaction-resolved descriptors could accelerate both conformational screening and selectivity prediction, provided they are grounded in the physically meaningful data. Within this framework, the ranked ensemble defines the subspace in which decisive noncovalent interactions operate; identifying and quantifying those interactions is the subject of the next section.

## 3 Atom- and Field-Resolved Dispersion

Once a physically meaningful low-energy conformational subspace has been defined, the central question becomes interaction-centered rather than structural: which interactions are responsible for the energetic discrimination between competing states?

Recent efforts to refine the language of noncovalent interactions have emphasized the importance of distinguishing geometric labels from the underlying physical components of interaction energies.^53^ In confined CIPs, disentangling the noncovalent interaction network requires quantitative, spatially resolved tools; among the contributing forces, London dispersion demands particular attention because its magnitude scales with the contact surface area enforced by the catalytic pocket. A compelling example is the revisitation of the Houk–List model,^54^ which revealed that dispersion influences stereoselectivity even in relatively compact organocatalytic transition states.^55^ In confined systems, the dense network of contacts within the catalytic pocket is expected to enhance the role of dispersion forces. This calls for quantitative tools that trace how dispersion emerges from molecular structure and influences selectivity.

A broad toolbox exists for analyzing noncovalent interactions, ranging from quantitative methods—such as Morokuma-type energy decomposition analyses (EDA^56–58^ ) and symmetry-adapted perturbation theory (SAPT^59^), which can disentangle the individual physical components of an interaction—to interpretation tools like the NCI index,^60^ which uses real-space features of the electron density to visualize weak attractive and repulsive contacts. In the following, we focus on tools available in the ORCA code^51^ that are most directly connected to the dispersion-centered perspective of this Account. At the correlated wave function level, the Local Energy Decomposition (LED) scheme provides rigorous partitioning of coupled-cluster interaction energies into physically meaningful contributions, such as electrostatics exchange, short-range repulsion and dispersion,^29,37,61^ all while maintaining DLPNO–CCSD­(T) accuracy. Owing to its unique combination of high accuracy and chemical interpretability, the LED scheme has been central to our research efforts, both in terms of method development and practical applications. A key challenge in this context is achieving functional-group—and ultimately atomic—resolution of dispersion contributions, which is essential for the prospective design of efficient catalytic systems. To this end, we introduced the Atomic Decomposition of London Dispersion (ADLD), formulated within both DFT^2^ and DLPNO–CCSD­(T)/LED^38^ frameworks. In this approach, the total dispersion energy is decomposed into atomic terms that collect all dispersion interactions involving a given atom. From these contributions, spatial LD densities (ρ_disp_) are constructed as real-space representations of atomic dispersion energies. Differences between such densities, which we call LD difference densities (Δρ_disp_), directly reveal how the dispersion associated with each atom changes upon structural reorganization.

In the asymmetric Diels–Alder reaction catalyzed by a BALT ion pair (Figure 3), this analysis revealed that the dominant dispersive bias arises from peripheral aryl substituents and distal scaffold regions. Although remote from the reactive center and marginal in electrostatic complementarity, these fragments accumulate stabilizing London dispersion contacts in the major transition state. The transition-state structures leading to the experimentally observed minor (**3a′**) and major (**3a**) enantiomeric products are shown in Figure 3a. The corresponding Δρ_disp_(r) functions illustrate which atoms contribute most significantly to the dispersion interaction between cyclopentadiene and the nucleophile–catalyst adduct in the respective transition states (Figure 3b). Atomic contributions can be recombined into functional groups or larger structural domains, thereby directly linking the dispersion bias to modular catalyst design (Figure 3c).

**Figure 3:**
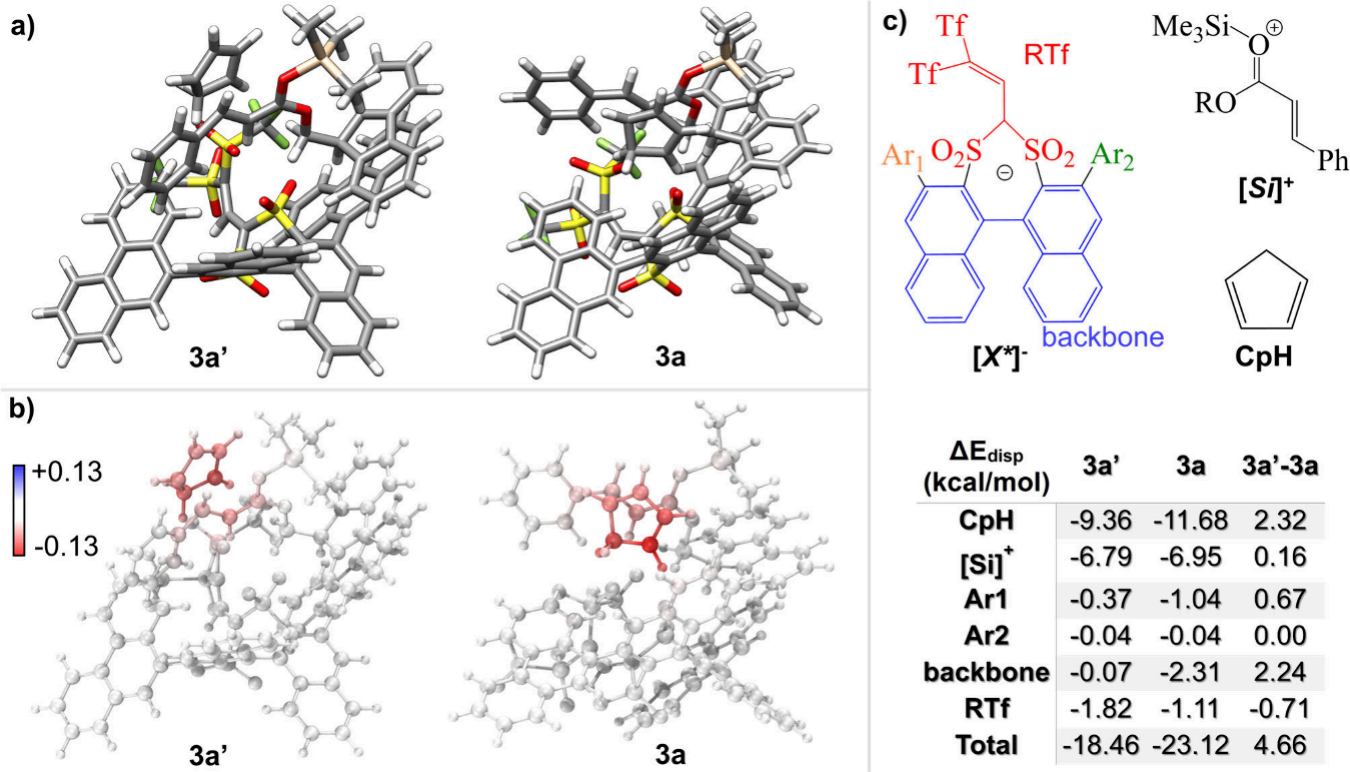
Atomic-level visualization of dispersion-driven enantiodiscrimination in an acid-catalyzed asymmetric Diels–Alder reaction. (a) Stereocontrolling transition-state structures leading to the major (**3a**) and minor (**3a′**) enantiomers. (b) Δρ_disp_ between the two TSs, highlighting regions more strongly stabilized in the major pathway. (c) Decomposition of Δρ_disp_ into contributions from individual functional groups, revealing how specific catalyst and substrate fragments differentially contribute to enantiomeric discrimination. Adapted with permission from ref 2. Copyright 2024 American Chemical Society.

Individual atoms—and the substituents and structural elements they constitute—thus become operational units whose differential contributions can be measured, ranked, and compared across competing pathways. In this way, ADLD addresses the need articulated by Schreiner for a quantitatively accurate scale of dispersion energy donors (DEDs) to guide rational molecular design.^34,63^

While ADLD identifies *which atoms* contribute to dispersion, a complementary question is *where in space* does dispersion accumulate and guide molecular recognition? Field-based descriptions address this aspect. For example, Pollice and Chen introduced the London dispersion potential (LDP) to map intrinsic dispersion propensity across molecular surfaces.^64^ Building on this concept, we developed the molecular dispersion potential (MDP),^3^ where dispersion is evaluated as the interaction between the system and a neutral probe (e.g., helium). Importantly, MDP and ADLD, being formulated at the same level of theory used for the electronic energies, from DFT-D3/D4 to DLPNO–CCSD­(T)/LED, allow dispersion to be analyzed within the same energetic framework that governs the reaction profile.

The integration of MDP with ADLD is also potentially extremely powerful: dispersion “hot regions” identified in the MDP can be traced back to the specific atoms responsible for generating that field feature through atomic decomposition. The two approaches thus provide complementary field- and atom-resolved projections of the same dispersion energy.

In extended supramolecular architectures, this dual perspective is especially valuable. MDP maps reveal anisotropic dispersion basins associated with extended π-surfaces and confined pockets, that can also be remote from the formal ion-pair center. Dispersion in fact extends beyond the catalyst–substrate interface, involving distal scaffold regions that engage secondary species such as solvent molecules. For example, in ref 3, MDP “hot regions” accurately predicted preferential catalyst–solvent interaction sites without exhaustive solvent sampling (Figure 4). These remote dispersive contacts can subtly rebalance transition-state stabilization and influence stereochemical outcome.

**Figure 4:**
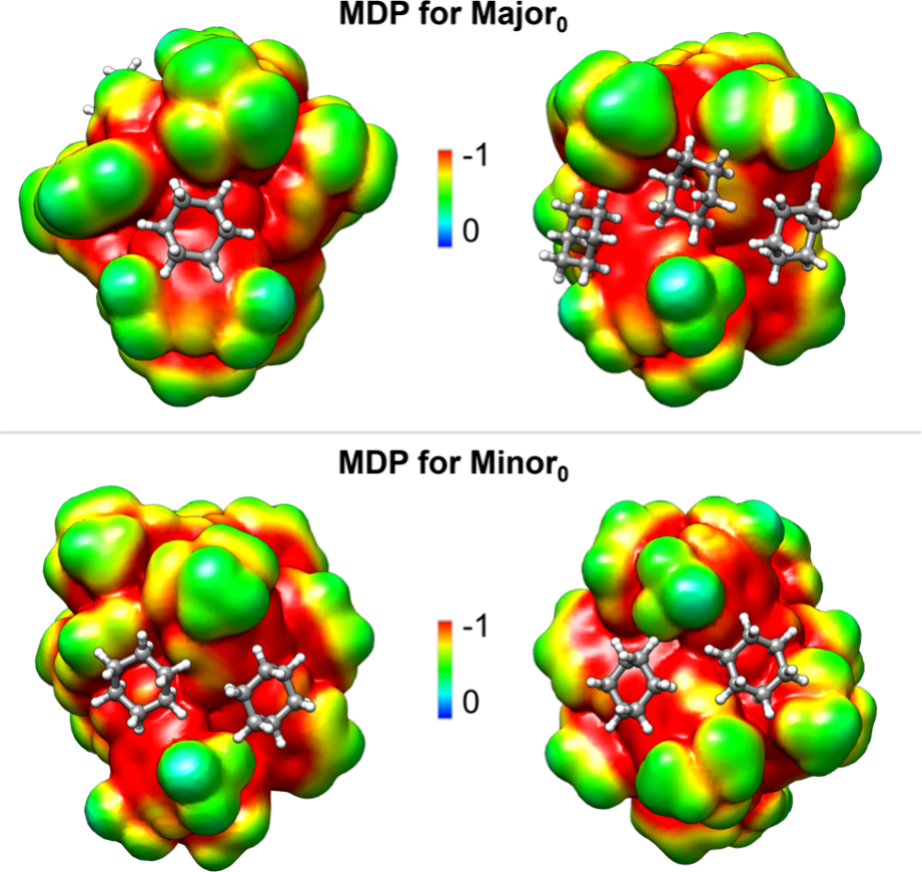
MDP for the most stable conformers of the major (**Major**_**0**_) and minor (**Minor**_**0**_) transition states. Cyclohexane molecules, resolved through microsolvation calculations, occupy the most attractive dispersive basins. This spatial correlation validates the MDP as a predictive descriptor for solvent localization around supramolecular catalytic assemblies. Adapted from ref 3 under CC BY 3.0.

## 4 Dispersion As a Design Lever for Selectivity

The stereochemical outcome of a confined asymmetric catalytic reaction is determined by three experimentally tunable components (the catalyst, the substrate, and the solvent), whose dispersive contacts are coupled: modifying any one redistributes the noncovalent network across the entire assembly. Because the catalyst cavity is where confinement originates, it provides a natural starting point for our discussion.

### 4.1 Catalyst

Dispersion is a *structural organizing force* that shapes how the reactive couple is steered along the productive pathways. A key concept emerging from our previous studies on these systems,^1,4,25^ which also extends across different catalytic platforms,^33,65,66^ is that modular catalyst fragments do not have an inherent “steric” or “attractive” character; rather, their role depends on the regime of the intermolecular potential at which they operate (Scheme 1).

**Scheme 1:**
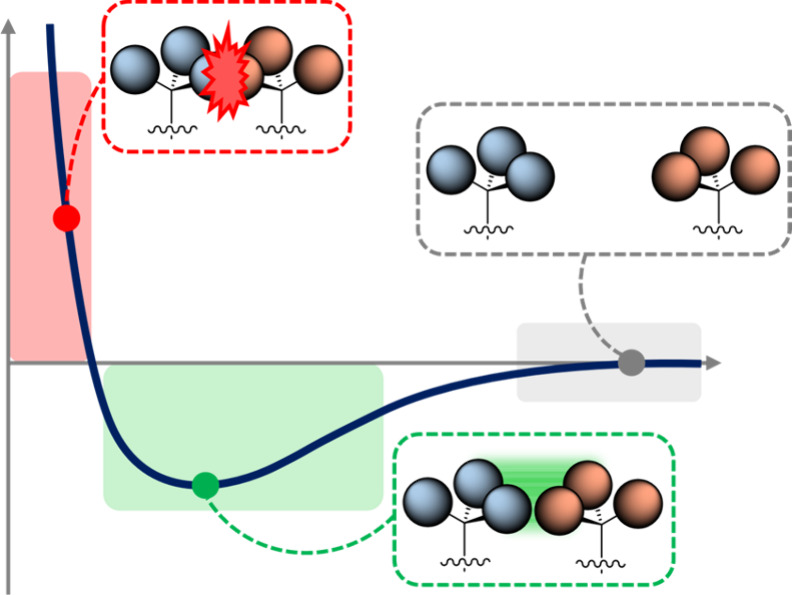
Distance-Dependent Intermolecular Potential between Two Fragments^a^

a Illustrates how the same modular unit can be repulsive, stabilizing, or negligible depending on separation. In modular catalysts, this enables systematic control of how fragments engage along the potential, providing a handle on stereochemical outcome.

For large, modular catalysts, this reframing offers a powerful interpretive and design lever: the contribution of each tunable fragment to enantiocontrol can be rationalized—and ultimately engineered—by identifying whether confinement positions it in the repulsive, attractive, or negligible regime of the potential. Within this picture, dispersion acts at multiple levels within the catalyst: (i) it sculpts preferential binding basins within the chiral pocket (recognition), (ii) it stabilizes specific intermediate and TS conformers within those basins (selection), and (iii) it enables mutual catalyst–substrate adaptation consistent with an induced-fit picture (response). The following examples illustrate these roles in complementary ways.

A clear illustration comes from our computational analysis of List’s asymmetric ion-pairing Diels–Alder chemistry, where BALT- and IDPi-based silylated Lewis acid organocatalysts promote enantioselective cycloadditions between cinnamate esters and cyclopentadiene.^1^ LED analysis reveals that, although nondispersive interactions favor the less congested approach, the more congested transition-state arrangement gains substantially stronger London dispersion stabilization. This additional dispersive contribution overcompensates steric repulsion and ultimately determines the observed enantioselection. This finding reinforces the view that bulky groups hinder or help depending on which regime of the van der Waals potential they operate in, and that within confined pockets enforcing contact at attractive distances, the same groups become dispersive stabilizers that can enhance reactivity and selectivity. The analysis explicitly framed dispersion as a *future design element* for IDPi catalysts, because substituent size/polarizability on the BINOL-derived framework modulates the pocket shape and the dispersive landscape that stabilizes CIP transition state structures.

This logic extends to the induced-fit case study on IDPi-catalyzed intramolecular hydroalkoxylation.^25^ Here, the stereochemical outcome is governed by a dispersion-driven induced-fit model in which the major pathway minimizes the geometric distortion penalty while maximizing catalyst–substrate attraction. Quantitatively, the activation barrier for the major pathway (20.0 kcal/mol) is essentially identical in magnitude to the thermal and entropic correction term, indicating that the geometric distortion penalty (+50.5 kcal/mol) is fully compensated by attractive interactions—of which London dispersion is the dominant component (−38.2 kcal/mol). Crucially, dispersion controls both sides of this balance: intramolecular dispersion within the catalyst governs the cost of reshaping the cavity, while intermolecular catalyst–substrate dispersion provides the principal stabilizing contribution at the transition state. Substituent deletion analyses sharpen this picture: in the major pathway, stabilizing intramolecular Ph/backbone dispersion contacts are preserved during pocket reorganization, whereas in the minor pathways these contacts are disrupted to accommodate alternative substrate orientations, increasing the distortion penalty. The design implication is that confinement alone does not guarantee selectivity; rather, the catalyst must retain sufficient flexibility to preferentially stabilize one substrate pose over competing alternatives.

The preceding case studies emphasize the role of dispersion at the transition state, but in confined catalysts its influence extends across the full reaction coordinate. In the ACPC radical [2 + 2] cycloaddition,^4^ the IDPi pocket already biases the reaction *before* the selectivity-determining TS, by acting as a dispersive *glue* between the anchored radical cation and the entering styrene. Productive approaches featuring π-stacked alignment of the reactive couple, with close olefin–olefin proximity, form stable encounter complexes, whereas approaches from the opposite face reorganize within the pocket into nonproductive arrangements that fail to maintain the reactive alignment required for bond formation (Figure 5a).

**Figure 5:**
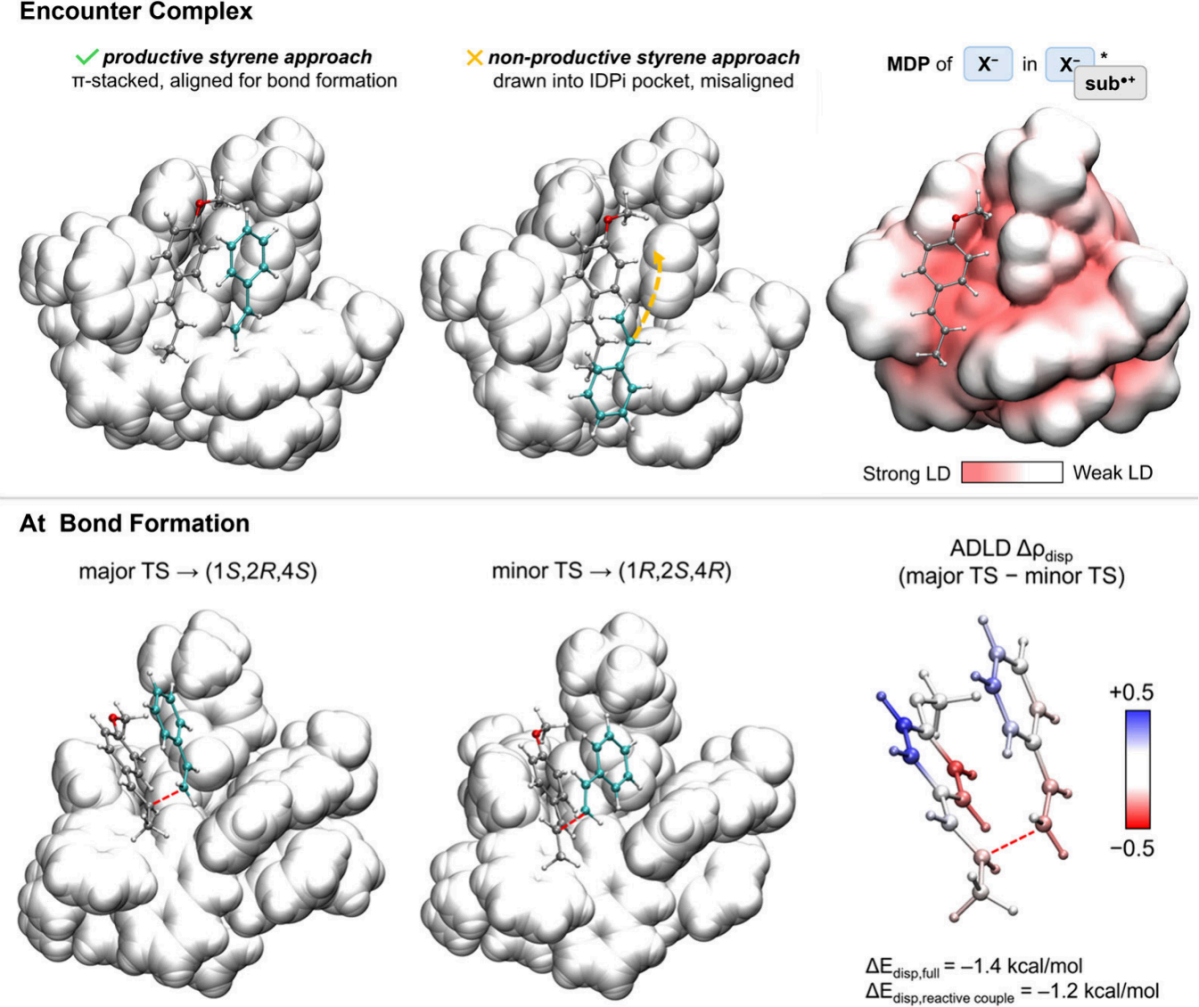
Dispersion-driven encounter-complex organization and transition-state selection in the IDPi-catalyzed ACPC radical [2 + 2] cycloaddition (radical cation C atoms in silver; styrene C atoms in cyan). Upper panel: Prereactive encounter complexes and MDP of the IDPi anion, showing the dispersion-rich pocket that stabilizes productive alignments while directing opposite approaches into nonproductive geometries. Lower panel: Transition-state structures leading to the major and minor enantiomers and ADLD dispersion density difference between them. Adapted from ref 4 under CC BY 4.0.

MDP analysis shows that the pocket’s dispersion field filters the accessible approaches and directs the system toward productive encounter complexes. Once there, dispersion differentiates between competing TSs. ADLD gives a ∼1.4 kcal·mol^–1^ dispersion difference between enantiomeric transition states, of which ∼1.2 kcal·mol^–1^ is localized on the atoms of the reactive couple itself. In this case, the catalyst provides a mostly rigid dispersion environment, and selectivity arises from how well the reacting partners maximize their mutual dispersion interactions—similar to a lock-and-key model mechanism, rather than the induced fit model described above.

Therefore, confined CIPs can access different canonical modes of enzymatic selectivity, from the original Fischer’s rigid lock-and-key complementarity^67^ to the Koshland’s refined induced fit model,^68^ the two frameworks that have jointly shaped our understanding of enzymatic recognition for over a century. This suggests that the design of catalyst architecture and the specific transformation it catalyzes may span the full continuum of recognition mechanisms that biology has evolved over billions of years.^69^ Having established how the catalyst pocket can encode, adapt, and filter dispersive interactions, we next consider how substrate modifications determine which parts of the pocket are actually engaged.

### 4.2 Substrate

If catalyst modularity provides the scaffold for stereocontrol, substrate modularity determines how that scaffold is populated. In CIPs, modifying the substrate reorganizes the entire network of noncovalent contacts within the confined pocket. As a result, small structural changes on the substrate can therefore reshape the balance of stabilizing and destabilizing contributions along the reaction coordinate, leading to measurable variations in ΔΔG^‡^.

In enzymology, Koshland^69^ recognized that substrate modifications change how catalytic groups are aligned and whether productive chemistry can occur. In our ACPC radical [2 + 2] cycloaddition, we observed a dispersive analogue of this principle: replacing hydrogen with a methyl group at the *para* position of styrene reduces enantiodiscrimination by just selectively strengthening dispersive contacts with the catalyst pocket in the disfavored pathway. ADLD helped in rationalizing the origin: the *para* substituent projects into the cavity exclusively in the minor TS (Figure 6), where it gains ∼0.6 kcal·mol^–1^ of additional dispersion stabilization, narrowing the free-energy gap from 2.3 to 1.8 kcal·mol^–1^—a trend consistent with the experimental erosion of ee across para-substituted styrenes.

**Figure 6:**
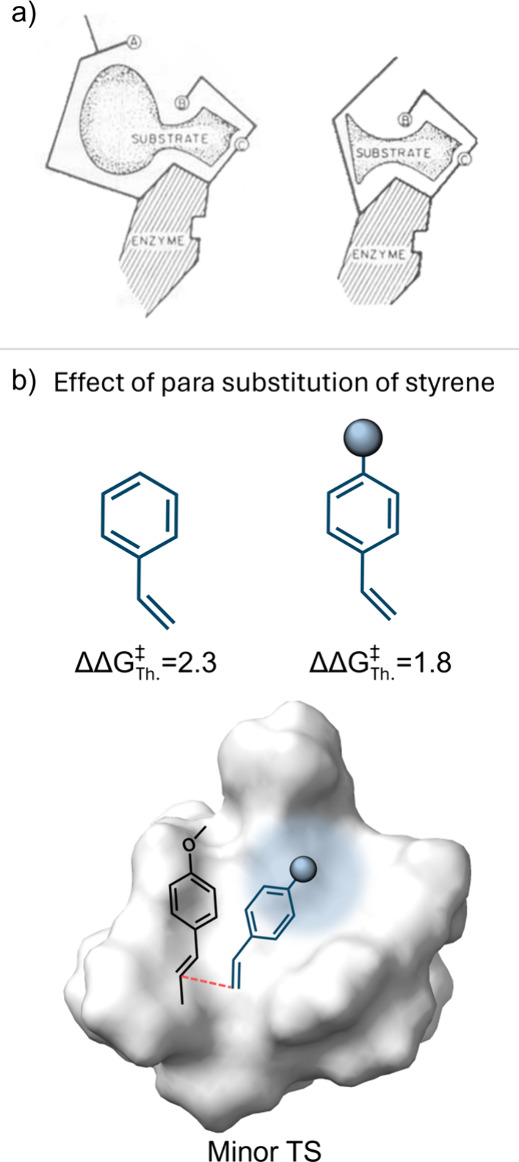
Substrate modifications reshape the interaction landscape within confined active sites. (a) In Koshland’s induced-fit framework, adding a bulky group to the substrate prevents proper alignment of catalytic groups A and B (left), while deletion of a group eliminates the buttressing action needed to maintain alignment (right). Adapted with permission from ref 69. Copyright 2003 John Wiley and Sons. (b) In the IDPi-catalyzed ACPC [2 + 2] cycloaddition, *para* substitution on styrene projects into the catalyst cavity only in the minor TS: the substituent lowers enantioselectivity by increasing dispersive contacts with the pocket.

The Mukaiyama–Michael reaction under IDPi catalysis^70^ provides further evidence that substrate groups remote from the reactive center can be deliberately exploited as dispersive recognition handles. ADLD analysis of the CIP preceding C–C bond formation reveals that the substrate’s pentyl side chain preferentially stabilizes the ion-pair arrangement with one catalyst variant over another, consistent with the experimentally observed reversal and amplification of enantioselectivity.

These examples establish substrate architecture as a programmable variable for enantioselectivity. Engineering substrate topology tunes the interaction pattern within the pocket as directly as modifying the catalyst scaffold, and rational optimization therefore requires a codesign strategy that treats catalyst and substrate as an integrated assembly. Importantly, once the catalyst–substrate interaction pattern is understood at atomic resolution, even seemingly peripheral substituents become actionable design elements. The same logic also makes clear why the surrounding medium cannot always be treated as a passive background: solvent molecules may compete for, reinforce, or disrupt the dispersive contacts encoded by the catalyst–substrate assembly.

### 4.3 Solvent

In computational catalysis, the solvent is often approximated as a continuum that screens electrostatics while leaving the underlying interaction network essentially unchanged. This approximation becomes increasingly fragile in confined supermolecular systems. However, explicit solvation dramatically increases configurational complexity: each additional solvent molecule introduces degrees of freedom that, coupled with the ones of the catalyst and the substrate, lead to combinatorial growth of accessible states. While a range of computational strategies have been proposed to try to address this challenge,^71–73^ capturing such effects accurately in confined catalysis remains a major challenge for theory.^3^

In particular, the role of solute–solvent van der Waals forces on transition-state ordering in asymmetric catalysis had remained largely unexplored. Our study on the IDPi-catalyzed hydroalkoxylation provided a first quantitative window into this phenomenon (Figure 4). Using a hybrid implicit/explicit microsolvation approach, we found that solvent–catalyst dispersion is not uniformly distributed but concentrated on specific catalyst fragments—the same aromatic and fluorinated groups that dominate intracatalyst dispersion. This creates an inherent tension: the solvent can in principle attenuate the intramolecular dispersive interactions that stabilize the catalyst’s preferred conformation, influencing the transition-state structural ensemble. For certain conformers, this redistribution meaningfully shifts their relative energies—an effect invisible to purely electrostatic solvation models. Notably, MDP maps of the unsolvated transition states correctly predicted the preferential solvent binding regions, validating the descriptor as a practical tool for anticipating where solvent may intervene. Solvent thus emerges as a third degree of freedom in confined asymmetric catalysis: solvent choice can in principle attenuate or amplify the stereochemical bias encoded by catalyst and substrate architecture.

## 5 Conclusions

The premise that has guided every stage of this Account is that reproducing experimental enantioselectivities, however accurately, is not an end in itself. The real payoff of computational catalysis lies in extracting transferable, interaction-resolved insights that connect molecular structure to function at the resolution required for molecular design and that can ultimately inform both human intuition and data-driven models. Confined CIPs have served as the testing ground for this philosophy: they combine enzyme-like structural complexity with the synthetic versatility of fragment-based design—large enough for noncovalent interactions to accumulate, modular enough for those interactions to be tuned, and confined enough for their effect on enantioselectivity to become decisive. The work examined here establishes that, in this regime, London dispersion is a chemically engineerable contributor to stereocontrol.

A recurring finding across all case studies is that the conventional separation between “steric” and “attractive” interactions breaks down inside a confined pocket. What has been rationalized for a long time as a steric effect is, at the electronic structure level, a composite of short-range repulsion and London dispersion whose balance shifts with every geometric rearrangement. In the examples presented here, the more congested transition state is often more stable, as short-range contacts generate dispersive stabilization that overcompensates repulsion. This reframing carries a practical message: molecular building blocks should be selected not only for the volume they exclude but also for the dispersive surface they provide. Moreover, enantioselectivity in confined systems cannot be attributed to any single component acting in the transformation. Catalyst, substrate, and solvent form a coupled interaction network in which structural modifications at any point redistribute contacts on which dispersion builds throughout the entire assembly. A compelling experimental illustration of this principle is provided by recent catalyst redesign in asymmetric transfer hydrogenation, where relocating large, polarizable groups from the reagent environment to the catalyst scaffold improved the scope and selectivity, consistent with the view that dispersion can act as a targetable design variable.^74^ The recurring parallels with enzymatic recognition mechanisms observed throughout this Account reinforce this perspective and suggest that the design space accessible to confined synthetic catalysts extends well beyond what has been explored thus far. Translating this understanding into practice, however, demands analytical tools that resolve dispersion at the scale at which catalysts are actually designed: atoms, functional groups, and modular scaffold regions. ADLD and MDP meet this demand by making the dispersion legible and quantifiable.

The open frontier, and ongoing challenge in our group, is to extend this resolution to the full noncovalent interaction network—steric repulsion, electrostatics, and dispersion expressed on the same energetic scale—so that their competition and cooperation within a confined pocket can be mapped with the same clarity that we have achieved here for dispersion alone. Looking forward, these physically interpretable descriptors may also provide a natural foundation for machine-learning models aimed at accelerating conformer selection, high-level energy ranking, and selectivity prediction without losing a direct connection to the underlying interaction network. Such a unified interaction language would help to complete the transition from post hoc rationalization to prospective design: encoding desired selectivity outcomes directly into molecular structure, before synthesis. If confined catalysis teaches us that function emerges from organized complexity, then London dispersion teaches us that even the most subtle molecular forces become decisive when that complexity is confined, modular, and resolved. Making these relatively weak forces visible, quantifiable, and engineerable is the path from mechanistic understanding to predictive molecular design.
